# Outcomes of CO_2_ laser-assisted posterior cordectomy in bilateral vocal cord paralysis in 132 cases

**DOI:** 10.1007/s10103-018-2478-9

**Published:** 2018-03-20

**Authors:** Joanna Jackowska, Elisabeth V Sjogren, Anna Bartochowska, Hanna Czerniejewska-Wolska, Krzysztof Piersiala, Malgorzata Wierzbicka

**Affiliations:** 1 Department of Otolaryngology, Head and Neck Surgery, Poznan University of Medical Sciences, Poznan, Poland; 2 Department of Otolaryngology, Head and Neck Surgery, Leiden University Medical Centre, Leiden, The Netherlands; 3 Department of Phoniatrics and Audiology, Poznan University of Medical Sciences, Poznan, Poland; 4 Student Research Group at Department of Otolaryngology, Head and Neck Surgery, Poznan University of Medical Sciences, Poznan, Poland

**Keywords:** Laser, Larynx, Vocal fold paralysis, Glottis

## Abstract

The purpose of the study was to assess the role of laser-assisted posterior cordectomy in the management of patients with bilateral vocal cord paralysis. We aimed an analysis of 132 consecutive patients treated by CO_2_ laser posterior cordectomy, aged 38–91, 31% tracheotomized on admission. Cordectomy was performed under microlaryngoscopy using CO_2_ laser (Lumenis AcuPulse 40 CO_2_ laser, wavelength 10.6 μm, Lumenis Ltd., Yokneam, Israel). We looked at the number of laser glottic procedures necessary to achieve decannulation in tracheotomized patients and to achieve respiratory comfort in non-tracheotomized subjects and we evaluated the two groups for differences in patient characteristics. In tracheotomized patients, we also assessed factors affecting the success of decannulation and we evaluated the impact of tracheotomy on patients’ lives. Decannulation was performed in 63% of tracheotomized patients. In terms of the number of procedures, 54% (14), 19% (5), and 27% (7) tracheotomized vs. 74% (61), 24% (20), and 2% (2) non-tracheotomized subjects underwent one, two, or three procedures, respectively. In the group of tracheotomized patients who were successfully decannulated, the number of multiple laser-assisted procedures was significantly higher than in the group of non-tracheotomized subjects with respiratory comfort after treatment (*p* = 0.04). Advanced age (> 66 years), comorbidities (diabetes, gastroesophageal reflux disease (GERD)), multiple thyroid surgeries, and tracheotomy below the cricoid cartilage were found to decrease the likelihood of successful decannulation. Posterior cordectomy is a simple method allowing for airway improvement and decannulation in patients with bilateral vocal cord paralysis. It is less effective in tracheotomized subjects with diabetes or GERD, older than 66 years old, after two or more thyroidectomies.

## Introduction

According to multicenter analyses, there are multiple reasons for bilateral vocal cord immobilization, but the prevailing reason, accounting for 26–59% of all reported cases, is bilateral vocal cord paralysis is iatrogenic due to surgery within the neck (thyroid, parathyroid glands, thymus, esophagus, carotid body paragangliomas) as well as cardio-, thoraco-, and neurosurgical procedures [1–7]. Prolonged or abnormal intubation is the cause of 1–31% of cases of vocal cord immobilization [1, 5, 8], whereas instrumental perinatal traumas are rare [9]. Thyroid surgery is the single most common cause of persistent iatrogenic bilateral cord paralysis and accounts for almost a quarter of all cases [5, 10–15]. The problem occurs in 1% of thyroidectomies; however, it is likely to be more frequent in thyroid cancer surgeries, retrosternal goiter, major intraoperative bleeding, and multiple thyroid surgeries (20–30%) [9, 14, 16, 17].

The clinical presentation depends on the extent of nerve injury (neuropraxis, axonotmesis, neurotmesis) and of the degree of residual laryngeal muscle innervation. Signs and symptoms are related to the three main functions of the larynx: airway, voice, and swallowing. Of these, airway impairment due to glottic narrowing is the most common problem and is considered the most dangerous. Paralysis of both vocal cords is usually manifested by stridor and/or dyspnea of varied intensity [7, 14, 17, 18] and calls for immediate emergency intervention, such as tracheotomy, intubation, or unilateral laterofixation, in approximately 50% of patients [5].

Until the late nineteenth century, the tracheotomy was the only surgical method to treat dyspnea resulting from bilateral vocal cord paralysis [8, 9]. This procedure dates back to ancient times and used to be the “gold standard” of respiratory tract protection [10]. However, tracheotomy is poorly accepted among patients; the majority of whom nowadays elect to undergo a glottic widening procedure such as laser-assisted posterior cordectomy to restore the glottic airway and allow for decannulation as soon as possible. These procedures result in permanent tissue loss and can be performed after 6–12 months, once it has been confirmed that the function of the laryngeal nerve cannot be restored.

The aim of this study was to assess the role of laser-assisted posterior cordectomy in the management of patients with bilateral vocal cord paralysis. We looked specifically at the number of laser glottic procedures necessary to achieve decannulation in tracheotomized patients and to achieve respiratory comfort in non-tracheotomized subjects and we evaluated the two groups for differences in patient characteristics. In tracheotomized patients, we also assessed factors affecting the success of decannulation and we evaluated the impact of tracheotomy on patients’ occupational lives.

Our hypothesis was that CO_2_ laser posterior cordectomy is a useful and successful treatment method in management of bilateral vocal fold paralysis and it helps in achieving prompt decannulation in affected subjects.

## Material and methods

**Fig. 1:**
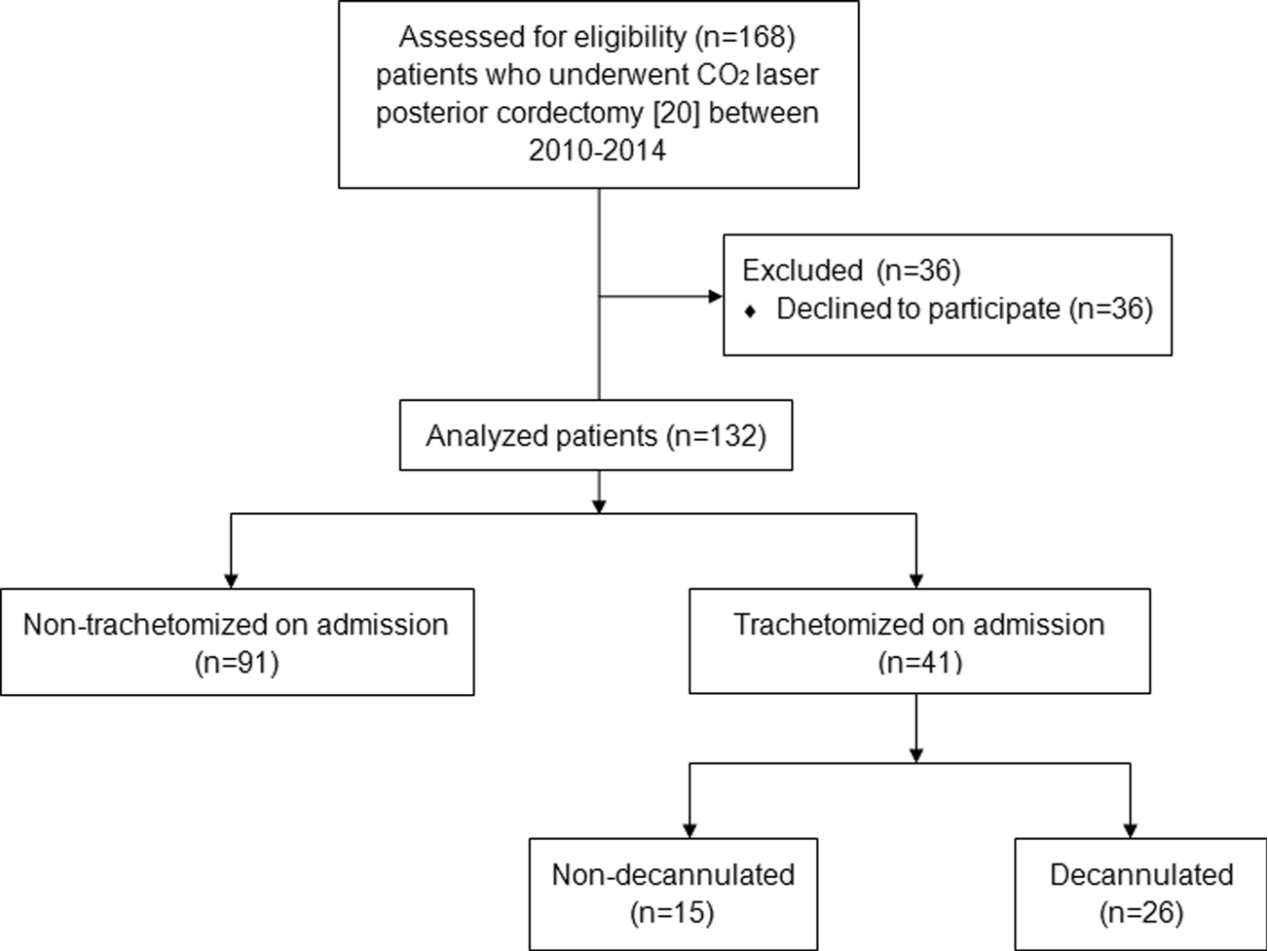
The study group

This study was conducted in a tertiary referral center, in 132 consecutive patients with bilateral vocal cord paralysis with a significant airway compromise admitted for CO_2_ laser posterior cordectomy as described by Dennis and Kashima [19] between 2010 and 2014 (Fig. 1).

To assess the role of laser-assisted posterior cordectomy in this patient cohort, we registered in how many patients the treatment was successful (decannulation in tracheotomized patients and respiratory comfort in non-tracheotomized patients). The number of procedures necessary to achieve this (1, 2, or 3 or more) was compared between the two groups using Student’s *T* test. The two groups were also compared in terms of the following characteristics: age, sex, body weight, number of previous thyroidectomies, time between vocal cord paralysis and referral for treatment, days of hospitalization, comorbidities, and coexistent subglottic stenosis using the Student *T* , Fisher, chi-square, and Mann-Whitney tests. Chi-square and Mann-Whitney tests were also used to assess the impact of these factors as well as tracheotomy technique on the possibility of decannulation in tracheotomized patients. Data tested with the Student *T* test were normally distributed. The significance level for all calculations was *p* < 0.05. Estimation of the power analysis was set at 80% including beta value at 20% for sample size in the case of mean differences using Student’s *T*, Fisher, chi-square, and Mann-Whitney tests. Statistical analysis was performed using Statistica 10 (StatSoft Polska, Kraków, Poland). Informed written consent was obtained from all patients. The protocol of the investigation has been approved by The Institutional Review Board of the Poznan University of Medical Sciences (No. 927/13).

### The preoperative work-up and treatment

On admission, every patient underwent trans-nasal flexible video-endoscopy (video processor with integrated LED light source, model CV-170 with HD, ENF-VH, Olympus Corp, Tokyo, Japan) and video-stroboscopic laryngeal examination before decision-making. All surgical procedures were performed under general anesthesia. Cordectomy was performed under microlaryngoscopy using CO_2_ laser (Lumenis AcuPulse 40 CO_2_ laser, wavelength 10.6 μm, Lumenis Ltd., Yokneam, Israel). The laser was used in SuperPulse™ mode with power tailored on the target structures (average 7 W). The depth of tissue penetration was 1 mm with a single burst of energy lasting 0.3 ms.

## Results

A total of 121 females (97.1%) and 11 males (8.3%) aged between 38 and 91 years (mean age 63 years) who underwent CO_2_ laser posterior cordectomy between 2010 and 2014 were analyzed. The reasons for bilateral paralysis were as follows: thyroidectomy—121 patients (91.7%) (first procedure: 94 (71.2%), re-thyroidectomy: 27 (20.5%)), idiopathic—6 patients (4.5%), post-intubation—2 patients (1.5%), and thoracosurgical procedures—3 patients (2.3%). On admission, 91 (68.9%) patients were breathing naturally, while 41 (31.1%) were tracheotomized. In the group of tracheotomized patients, the mean time from vocal cord paralysis to tracheotomy was 3.2 years but ranged from 1 day to 22 years. The time between paralysis and the decision in favor of a laser-assisted glottic widening procedure ranged from 6 months to 23 years.

The first aim was to determine the proportion of treatment success in tracheotomized vs. non-tracheotomized patients by comparing the number of laser glottic procedures necessary to perform decannulation in tracheotomized subjects and to achieve respiratory comfort in non-tracheotomized subjects. In 15 tracheotomized patients (36.6%), decannulation was not possible and 8 non-tracheotomized patients (8.8%) did not reach respiratory comfort (*p* = 0.01). The remaining patients in whom treatment was successful were classified into three categories, depending on the number of surgical interventions before decannulation/respiratory comfort was reached: one, two, or three or more. In terms of the number of procedures, 54% (14), 19% (5), and 27% (7) tracheotomized vs. 74% (61), 24% (20), and 2% (2) non-tracheotomized subjects underwent one, two, or three procedures, respectively. In the group of tracheotomized patients who were successfully decannulated, the number of multiple laser-assisted procedures (two or more) was significantly higher than in the group of non-tracheotomized subjects with respiratory comfort after treatment (*p* = 0.04).

The second aim was to compare tracheotomized and non-tracheotomized patients with regard to several characteristics (Table 1). Tracheotomized patients were older than non-tracheotomized subjects (*p* = 0.003). There were no significant differences in the duration of a single hospitalization after laser treatment between tracheotomized (mean 3.8 days, median 3 days) and non-tracheotomized patients (mean 3.5 days, median 3 days) (*p* = 0.733). The occupational activity of patients with vocal cord paralysis after laser treatment was considered a vital factor in the assessment of the treatment outcome and was compared between tracheotomized and non-tracheotomized patients. After laser treatment, 20 patients (who could not work before laser surgery) resumed work: 3 tracheotomized and 17 non-tracheotomized subjects respectively. Almost twice as many tracheotomized patients (83%) failed to return to work after laser treatment as compared to non-tracheotomized subjects (45%). This correlation was statistically significant (*p* = 0.016).

**Table 1:** Comparison of tracheotomized and non-tracheotomized patients

	Non-tracheotomized (91)	Tracheotomized (41)	*p*
Age (in years)			0.003
Range	38–83	44–91	
Median	57	63	
Mean	58.6	62	
Sex			0.171
Female	81 (89%)	40 (98%)	
Male	10 (11%)	1 (2%)	
Comorbidities			
Diabetes	19 (21%)	6 (15%)	0.398
GERD	6 (6.5%)	2 (2%)	0.409
Other (cardio-vascular diseases)	18 (20%)	5 (12%)	0.290
Body weight			0.533
< 70 kg	61 (67%)	11 (26%)	
> 70 kg	30 (33%)	30 (74%)	
Time between vocal fold paralysis to referral for treatment (in years)			0.833
Range	0.5–20	0.5–23	
Median	2.0	2.0	
Mean	4.8	5.2	
Number of thyroidectomies	(81 subjects)	(40 subjects)	0.972
One	63 (78%)	31 (78%)	
More than one	18 (22%)	9 (22%)	
Hospitalization length after laser posterior cordectomy (in days)			0.733
Range	1–16	1–11	
Median	3	3	
Mean	3.5	3.8	
Subglottic stenosis	2 (2%)	8 (20%)	0.118

Finally, we investigated the factors that affected successful decannulation in patients tracheotomized due to bilateral cord paralysis (Table 2). Forty-one (41) subjects with tracheotomy were divided into two subgroups: patients with successful decannulation (26 patients; 63.4%) and patients in whom decannulation was not possible (15 patients; 36.6%). Time to successful decannulation in tracheotomized patients ranged from 5 to 150 days (mean 42, median 35 days). The mean and median age of decannulated vs. non-decannulated patients was 59.7 and 58, and 67.7 and 66 years, respectively. There was a correlation (*p* = 0.014) between age and decannulation outcome; the younger the patient on admission, the higher the chances of restoring the natural respiratory route. The number of previous thyroidectomies (one or more thyroid surgeries) was also found to affect the potential for decannulation. Of the 26 decannulated subjects, 23 (88%) had one thyroid surgery, and three (12%) patients had undergone two thyroid surgeries. Of the 15 non-decannulated patients (37.5%), nine (60%) had one thyroidectomy, and six (40%) had two or more. Thyroid reoperations were therefore shown to significantly decrease the possibility of tracheotomy withdrawal (*p* = 0.048). When examining tracheotomy technique, tracheotomy was considered to be correct (located between tracheal ring II and IV) in 34 (83%) out of 41 patients and was found to be too high (directly under the cricoid cartilage) in seven (17%) subjects. Decannulation was possible in 24 (92%) patients with correct tracheotomy and in 2 (8%) with high tracheotomy; the correct technique was directly associated with the probability of successful decannulation after laser surgery (*p* = 0.042). If the tracheotomy is too high, the likelihood of subglottic stenosis is significantly higher, which results in a clinically difficult situation of combined stenosis: paralytic at the glottic level and scar-like in the subglottic area. Out of 41 tracheotomized patients, 26 were ultimately decannulated: three out of eight patients with subglottic stenosis were successfully decannulated, whereas five remained permanently tracheotomized. Subglottic stenosis was confirmed in 33% of non-decannulated patients and in only 11% of decannulated patients; however, the difference was statistically insignificant (*p* = 0.117). As for the correlation between comorbidities, such as diabetes, gastroesophageal reflux disease (GERD), or hypothyroidism, and the likelihood of successful decannulation in the analyzed group, six subjects had diabetes, five of whom were decannulated (19%) and one non-decannulated (7%). Among the five patients with hypothyroidism, one (19%) was decannulated. GERD, confirmed by a pH assay, was noted in two decannulated and one non-decannulated patient. Additional comorbidity was found to adversely reduce the likelihood of future decannulation (*p* = 0.03). Independent breathing without a tracheotomy tube was achieved in only 38% patients with comorbidities. On the other hand, 62% of patients not treated for any of the aforementioned metabolic diseases were successfully decannulated. To study the correlation between body weight and successful decannulation, patients were divided into two subgroups: below 70 kg and above 70 kg body weight. The rate of decannulated patients with body mass below 70 kg was 73% compared to 40% of non-decannulated patients. In the group of patients with a body weight over 70 kg, these proportions were reversed—as many as 60% of patients still used a tracheotomy tube to breathe and only 27% of patients used the natural respiratory route; this difference was statistically insignificant (*p* = 0.148). Occupational activity was also investigated among tracheotomized patients divided into successfully decannulated and (7/11) non-decannulated (2/6) subjects. A total of 67% of non-decannulated patients and only 36% of decannulated patients failed to return to work after laser treatment; however, the difference was not statistically significant (*p* = 0.335). Patients were also asked to answer YES or NO in response to whether decannulation influenced their satisfaction with laser treatment: 24/26 decannulated (92%) subjects were satisfied.

**Table 2:** Comparison of parameters in decannulated and non-decannulated patients

41 patients with tracheotomy	Decannulated (26)	Non-decannulated (15)	*p*
Age (in years)			0.014
Range	44–75	50–91	
Mean	59	67	
Median	58	66	
Duration of tracheotomy (in years)			0.892
Range	0.5–23	0.5–21	
Mean	4.9	5.7	
Median	1.9	2.0	
Location of tracheotomy			0.042
Correct	24 (92%)	10 (67%)	
Too high	2 (8%)	5 (33%)	
Number of thyroidectomies			0.048
One	23 (88%)	9 (60%)	
More than one	3 (12%)	6 (40%)	
Presence of subglottic stenosis	3 (11%)	5 (33%)	0.117
Comorbidities	1 (4%)	10 (67%)	0.030
Body weight			0.148
< 70 kg	19 (73%)	6 (40%)	
> 70 kg	7 (27%)	9 (60%)	

## Discussion

The degree of dyspnea in patients with bilateral vocal cord paralysis depends on the width of the glottis. Severe dyspnea is observed at a critical stenosis below 0.5–1 mm; 1.5 mm allows for effective breathing at rest; however, dyspnea develops at low-intensity effort or during infection. The difference in intensity and onset of symptoms depends on the position and changes in the tension of vocal cords [14]. Immediately after the laryngeal nerve is cut, the vocal cords are positioned medially, are abducted laterally from the medial line by 2–4 mm, and are quite flaccid [14, 18, 20]. This creates enough space for ventilation in the posterior glottis segment; however, the contact and tension of the cords in the anterior glottis is not sufficient to provide a voice of appropriate quality [18]. After some time, the duration of which differs in each individual (even longer than 20 years like in our study group), cords become stiff, tension increases, and there is a shift to the level of the medial line of the larynx. At this point, the voice improves, along with dyspnea accompanying the smallest effort [14, 18, 21]. Medialization of the vocal cords depends on the shortening of the paretic vocal muscle due to atrophy and fibrosis and the activity of the cricothyroid muscle that is not paretic [6, 18].

Majority of patients with bilateral vocal cord paralysis have to undergo some form of surgical procedure to restore a patent airway [8]. Laser posterior cordectomy is always a compromise between effective respiration and preserving good voice quality, although the authors advocate that phonation is of secondary importance. Respiratory comfort without a tracheotomy is the treatment goal. In tracheotomized patients, the rate and time of decannulation is the most important indicator of treatment efficiency. Using a typical posterior Dennis and Kashima cordectomy, Ferri et al. [22] (9 patients) and Reker and Rudert [12] (6 patients) reported 100% decannulation within 3 to 60 days. Manolopoulos et al. [23] (18 patients) and Segas et al. [24] (20 patients) reported rates of 88.8 and 90%, respectively. Previously published data for partial arytenoidectomy or its combination with posterior cordectomy indicate even higher rates of successful decannulation. Remacle et al. [25] decannulated 100% out of eight tracheotomized patients between 10 and 15 days after the procedure, and Bizakis et al. [26] (18 patients) Maurizi et al. [9] (7 patients) all within 1 to 4.5 weeks. Motta et al. [27] performed partial arytenoidectomy with posterior cordectomy and ventricle resection in a group of 59 subjects; decannulation was possible in all 32 tracheotomized subjects after 7–15 days, although six (7.2%) patients required surgical revision. Ventriculocordectomy made it possible to restore the natural respiratory route in 76% of tracheotomized patients out of 41 presented by Pia et al. [28] and 88.2% out of 22 patients treated by Shvero et al. [1].

In our own study on a group of 132 patients with bilateral paralysis treated by posterior cordectomy according to Dennis and Kashima, we reached respiratory comfort in 83 out of 91 non-tracheotomized (91%), and 26 out of 41 tracheotomized subjects were decannulated (63%). The time to decannulation ranged from 5 to 150 days (mean 42 days, median 35 days). Decannulation was possible after one procedure in 14 (54%) patients, after two procedures in five patients (19%), and after at least three procedures in seven subjects (27%). The factors that affected successful decannulation were carefully analyzed. Advanced age, comorbidities, a higher number of re-thyroidectomies, and “high” tracheotomy, i.e., just below the cricoid cartilage, were shown to significantly reduce the probability of decannulation. Tracheotomized subjects had to undergo a statistically higher number of laser procedures to widen the glottis as compared to non-tracheotomized subjects in order to restore natural breathing.

The effects of tracheotomy on the future life of patients were also addressed. In a group analyzed by Segas et al. [24], tracheotomy was intentionally performed in 20 patients to save them from episodes of laryngeal dyspnea, despite being aware of the potential success with laser posterior cordectomy. In the present study group, tracheotomy was found to be a negative factor. Although the tracheotomy did not affect the time to decision regarding laser widening of the glottis, the general level of treatment satisfaction was significantly lower in non-decannulated patients as compared to both decannulated and non-tracheotomized subjects. Tracheotomy also significantly reduced the chances of returning to work, even if the patient was eventually decannulated.

## Conclusions

In conclusion, laser posterior cordectomy is a simple method allowing for airway improvement and decannulation in a majority of patients with bilateral vocal cord paralysis. It seems to be less effective in tracheotomized subjects, especially those with diabetes or GERD, older than 66 years old, after two or more thyroidectomies, or with subglottic stenosis due to “too high” tracheotomy.
